# Cellular senescence, neuroinflammation, and microRNAs: Possible interactions driving aging and neurodegeneration in the hippocampal neurogenic niche

**DOI:** 10.1016/j.nbas.2025.100141

**Published:** 2025-06-12

**Authors:** O. Polzer, E. Kinloch, C.P. Fitzsimons

**Affiliations:** Brain Plasticity Department, Swammerdam Institute for Life Sciences, Faculty of Science, University of Amsterdam, SciencePark 904, 1098XH Amsterdam, the Netherlands

**Keywords:** Senescence, Neuroinflammation, Neural stem cells, Adult neurogenesis, microRNA

## Abstract

Cellular senescence influences normal physiology and ageing-related diseases, including neurodegeneration. Senescent cells accumulate with age in the brain, secreting pro-inflammatory factors that promote neuroinflammation, which has been linked to disorders like Alzheimer’s and Parkinson’s. Neurons and other brain cells such as microglia, astrocytes, and neural stem/progenitor cells (NSPCs), exhibit senescence in aged brains. NSPCs, essential for neurogenesis, may enter senescence due to inflammatory signals and other factors and microRNAs may regulate this process. Here we discuss senescence mechanisms, neuroinflammation, and potential therapeutic targets, proposing that modulating senescence by microRNA-mediated pathways could help combat neurodegenerative diseases.

## Highlights

• Cellular senescence impacts normal physiology and ageing-related diseases.
• Neurons and neural stem and progenitor cells show senescence in aged brains.
• microRNAs may influence stem cell ageing and regeneration.
• microRNAs may help modulate senescence in neurodegenerative diseases.

## Introduction

Adult neural stem/progenitor cells (NSPCs) are long-lived somatic stem cells that predominantly remain in a quiescent state throughout adult life. This non-proliferative state, while protective, may make them susceptible to entering cellular senescence – a state of irreversible cell cycle arrest that impairs cell function and leads to a pro-inflammatory secretory profile. Interestingly, ablation of senescent cell from the NSPC niche in the murine hippocampus restores their proliferative and neurogenic capacity, thereby enhancing hippocampus-dependent spatial memory, supporting the concept that senescent cells negatively impact on NSPC function in the ageing brain [39]. NSPCs must be able to dynamically transition between quiescence and activation in response to various signals to maintain neurogenesis. However, with age, this dynamic process can be impaired and the likelihood of NSPCs entering long-term non-proliferative state increases. Understanding the precise regulation of the balance between different non-proliferative NSPC states may offer new avenues to restore/promote NSPC quiescence and reduce senescence in the ageing brain. Emerging evidence highlights microRNAs (miRNAs) as crucial post-transcriptional regulators in adult NSPCs which have been shown to target several senescence pathways [19,105]. Here, we discuss senescence definitions, the effect of senescence on the cellular microenvironment and its contribution to neuroinflammation. Then, we focus on NSPC senescence in the ageing brain, and the regulatory role of miRNAs in this process. Most of the literature we review concerns experimental evidence obtained from animal models. As the presence of ongoing neurogenesis in the adult human hippocampus remains under intense scrutiny, evidence from animal models can provide insightful mechanistic information but a direct applicability to humans might be speculative [44].

### *What is cellular senescence?*

Senescence is a cellular state characterised by irreversible cell cycle withdrawal and the production of a complex secretory phenotype known as the senescence-associated secretory phenotype (SASP) [60,138]. This ubiquitous process occurs in organisms from early life to adulthood and is important for tissue development and homeostasis. While senescence can have beneficial physiological functions in embryonic development, wound healing, and tumour suppression, excessive and aberrant accumulation of senescent cells can deteriorate the regenerative capacity of adult tissues and modulate their inflammatory environment [100]. It is these properties of senescent cells which may contribute to ageing and various age-related diseases.

Senescence can be distinguished from other non-proliferative cell states such as quiescence, by the cell’s irreversible arrest in G1 or G2 phases even in the presence of mitogenic stimuli. In quiescence, the cell cycle arrest in G0 phase is reversible and in most cases triggered by scarcity of nutrients and growth factors or long-term preservative factors [32,50,124,157].

Senescence can be classified as acute or chronic, according to its function and duration [133]. Acute senescence takes place during an organism’s normal developmental and homeostatic processes [100]. In acute senescence, specific cells are targeted and are quickly cleared by the immune system [133]. Contrarily, chronic senescence occurs in response to prolonged exposure to exogenous and endogenous factors such as telomere shortening and oncogene activation [51,82]. Chronic senescence is closely linked to ageing and age-related pathologies such as neurodegenerative disease. Over time, the accumulation of macromolecular damage exacerbates cellular stress, driving senescence in cells in a widespread, non-targeted manner while evading natural clearance mechanisms [51,96,133]. Within tissues, this chronic, extended senescence leads to inflammation, immunosenescence, and a loss of tissue function and regenerative potential. These features of senescence may lead to ageing and disease. Indeed, clearance of senescent cells within animal models extends lifespan and reduces occurrence of neurodegenerative diseases [9,11,16,147,153] .

The concept of senescence in the brain is more complex. Since neurons do not replicate, the key hallmark of cell cycle arrest becomes redundant for neurons [54], but not for other cell types that retain proliferative capacity in the adult brain. However, recent research indicates that neurons still undergo senescence, driven by age-related stress such as DNA damage, oxidative stress and oncogenic signalling [54], [160], [161], [162].

### Common hallmarks of cellular senescence

An accurate identification and classification are crucial for a clear understanding of the role of senescent cells in physiology and pathology. However, up to now, this task has proven to be challenging for two main reasons: 1) the phenotype of senescent cells changes over time and can be different depending on the cause and the cell type studied, 2) senescence-associated molecular and morphological changes are not exclusively observed in senescent cells but can be present in other non-senescent cell states and conditions. For these reasons, the guideline is to use a combination of biomarkers to more reliably identify senescent cells [95]. These range from traditional markers such as senescence associated β-galactosidase activity, cell cycle arrest markers and cell morphology changes to components of the complex and heterogeneous SASP. The SASP is the major mediator of the paracrine effects of senescent cells in their tissue microenvironment and is generally composed by cytokines, growth factors and proteases [141], emerging as a complex but key hallmark of senescent cells.

**Fig. 1:**
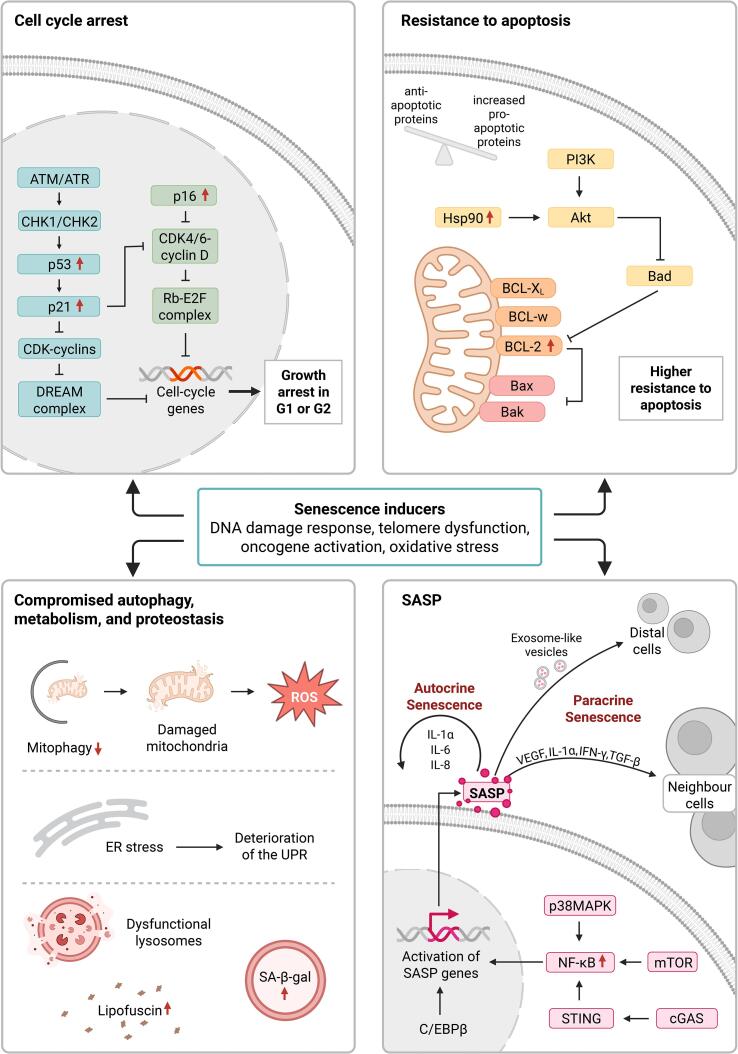
**Characteristics and mechanisms of cellular senescence.** Cellular senescence can be triggered and reinforced by various mechanisms including but not restricted to the DNA damage response (DDR), telomere dysfunction, oncogene activation and oxidative stress. Some senescence stressors lead to activation of the p53-p21 and the p16-Rb pathway. Both pathways result in decreased transcription of cell cycle genes and subsequent irreversible cell cycle arrest. Senescent cells display other common characteristics, including resistance to apoptosis, where they shift towards a pro-survival state. Furthermore, mitochondrial dysfunction, ER stress, accumulation of lysosomes, increase of lipofuscin and an increase in β-galactosidase (SA-β-gal) activity is observed. Another characteristic of senescent cells is the senescence-associated secretory phenotype (SASP), consisting of an amplitude of different cytokines, chemokines, growth modulators, angiogenic factors and matrix metalloproteinases (MMPs). Activators of the SASP include nuclear factor kappa B (NF-κB), p38 mitogen-activated protein kinase (p38MAPK), CCAAT/enhancer binding protein β (c/EBPβ), the cyclic GMP-AMP synthase (cGAS)- stimulator of interferon genes (STING) pathway, and mammalian target of rapamycin (mTOR). Reinforcement of the senescence programme can take place in an autocrine (via factors such as IL-1α, IL-6, and IL-8) or paracrine (via factors like VEGF, IFN-γ, TGF-β, IL-1α or exosome-like vesicles) manner. (ATM, ataxia-telangiectasia mutated; ATR, ataxia-telangiectasia and Rad3-Related protein; CHK1, checkpoint kinase 1; CHK2, checkpoint kinase 2; p53, Tumor protein p53; p21, cyclin-dependent kinase inhibitor 1A; CDK, cyclin-dependent kinase; DREAM complex, dimerization partner, RB-like, E2F and multi-vulval class B complex; p16, cyclin-dependent kinase Inhibitor 2A; CDK4/6, Cyclin-Dependent Kinase 4/6; Rb-E2F complex, retinoblastoma protein-E2F transcription factor complex; PI3K, phosphoinositide 3-kinase; HSP90, heat shock protein 90; Akt, AKT serine/threonine kinase; Bad, BCL2 Associated Agonist of Cell Death; Bcl-Xl, BCL2 Like 1; Bcl-w, BCL2 Like 2; Bcl-2, B-Cell Lymphoma 2; Bax, BCL2 Associated X; Bak, BCL2 Antagonist/Killer 1; ROS, Reactive Oxygen Species; ER, Endoplasmic Reticulum; UPR, Unfolded Protein Response, IL1α/γ/6/8, interleukin 1α/ γ /6/8; VEGF, vascular endothelial growth factor; TGF-β, Transforming Growth Factor Beta). Figure Created in BioRender. Fitzsimons, C. (2025) https://BioRender.com/d62c352.

Furthermore, cellular senescence is driven by various distinct and overlapping molecular pathways. Several cell stressors can drive and reinforce senescence in a cell and trigger the activation of these senescence-related signalling pathways. The most predominant cellular mechanisms leading to senescence, include the DNA damage response (DDR), telomere dysfunction, nuclear changes and oxidative stress [28,29,53,128]. These mechanisms contribute to the defining features of senescent cells, such as a loss of proliferative potential due to cell cycle arrest, decreased propensity to undergo apoptosis, increased metabolic activity, alterations in proteostasis, and elevated production of various secretory molecules into a SASP (Fig. 1).

### Cell cycle arrest

**Fig. 2:**
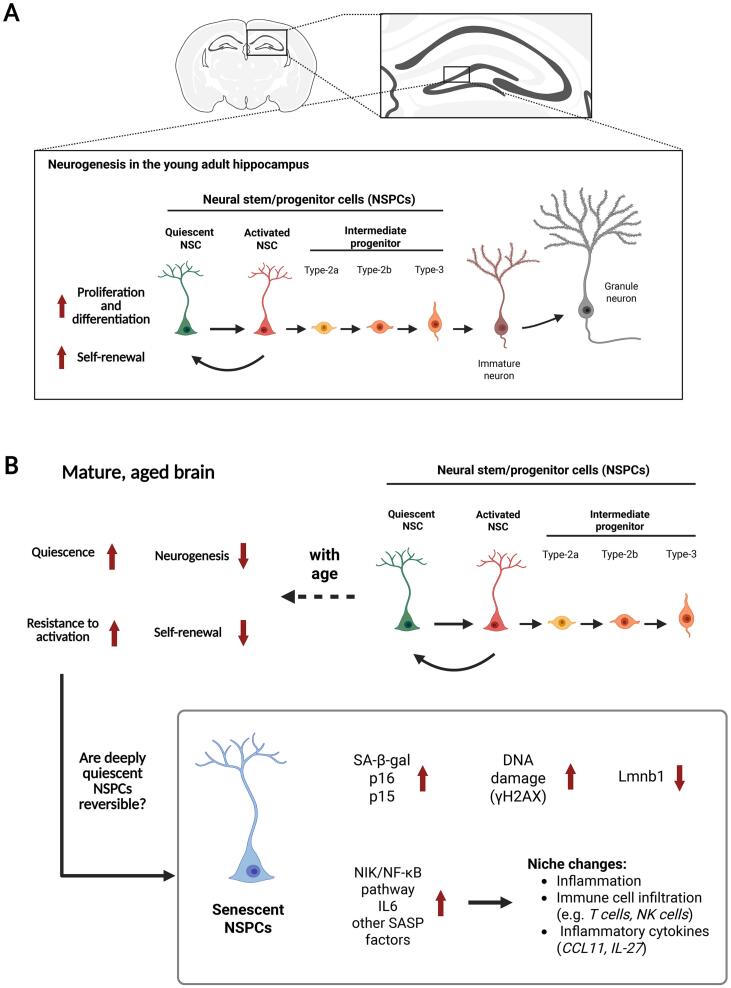
**Adult hippocampal neurogenesis in young and aged adult brains.** In the young brain (**A**) quiescent neural stem/progenitor cell (NSPC) can be activated to proliferate, differentiate and self-renew, supporting adult hippocampal neurogenesis. Whereas, in the aged brain (**B**) quiescence and the resistance in activation of adult NSPCs increases, leading to decrease neurogenesis and self-renewal. Aged NSPCs may undergo senescence, displaying an increase in characteristic senescence markers as senescence-associated secretory phenotype (SASP) factors. These factors likely contribute to a pro-inflammatory shift in the niche, further driving the infiltration of systematic innate and adaptive immune cells. This pro-inflammatory environment of the hippocampus may promote the remaining quiescent NSPCs to be locked in a deep quiescent state that is resistant to activation and subsequently reduces the brain’s capacity for adult hippocampal neurogenesis. Figure Created in BioRender. Fitzsimons, C. (2025) https://BioRender.com/r14q876.

As mentioned before, a crucial characteristic of senescent cells is irreversible cell cycle arrest. For a cell to undergo cell division, it requires the transcription and translation of cell cycle genes that mediate progression through the cell cycle. In senescent cells, stimuli such as DNA damage, epigenetic alterations or oxidative stress promote the activation of one or both of the p53-p21 and p16-Rb signalling pathways [104,107]. P21^CIP1/WAF1^ (p21) and p16 are cyclin dependent kinase inhibitors (CDKis) that block the transcription of cell cycle genes limiting cell cycle progression. P21 inactivates all cyclin-dependent kinases (CDKs) by forming the Drosophila RBF, E2f2 and Mip (DREAM) complex, which is a master repressor of cell cycle gene expression [110]. Therefore, when the p53/p21 signalling pathways are activated, the DREAM complex represses the transcription of cell cycle genes [72]. Similarly, p16 directly binds to CDK4/6 and inhibits the formation of cyclin D-CDK4/6 complexes, preventing the phosphorylation of Rb and promoting the formation of the repressive Rb-E2F complex [41]. The Rb-E2F complex binds to the promotor of cell cycle genes and reduces their transcription [115]. Due to the increased engagement of the CDKis, senescent cells are arrested in the G1 or G2 phase of the cell cycle and are unable to proliferate [32,50]. Prolonged expression of p53, activated Rb, p16 or p21 is sufficient to induce senescence [72,84]. Interestingly, recent observations indicate that, under pathological conditions, p21 could link cell senescence and cell quiescence, a process that we will discuss further later in this review [20]. DNA damage and the associated persistent DDR are strong anti-proliferative signals. If the damage to the DNA can be resolved, the cell re-enters the cell cycle. On the other hand, if the DNA damage cannot be repaired cells can become senescent, switching from a temporary into an indefinite growth arrest termed senescence-associated growth arrest (SAGA), which has also been detected in quiescent cells [120]. See (Fig. 2).

### Resistance to apoptosis

The ability of cells to bypass apoptosis in response to stress is essential in orientating the cell’s fate towards a senescent phenotype. Senescent cells undergo a significant shift towards pro-survival states through activation of anti-apoptotic B-cell lymphoma 2 (Bcl-2) factors and phosphatidylinositol 3-kinase (PI3K)/protein kinase B (AKT) signalling pathways. The Bcl-2 family of anti-apoptotic proteins are responsible for sequestering pro-apoptotic proteins and enabling cell survival [65]. In senescent cells, anti-apoptotic proteins are expressed at high levels compared to normal cells, thus disrupting the balance between anti- and pro-apoptosis [35]. This takes place at the transcriptional, translational, and epigenetic level [22,55]. Additionally, pro-apoptotic members of the Bcl-2 family may be downregulated in senescent cells [120]. For example, in senescent cells Bcl-w and Bcl-XL are translationally upregulated and the pro-apoptotic Bax gene is repressed due to enrichment with the histone mark H4K20me3 [55,113,150]. The PI3K/AKT signalling pathway further promotes cell survival in senescent cells by inhibition of pro-survival BH3-only protein BAD and upregulation of MCL-1. Moreover, heat shock protein 90 (Hsp90) is over-active in senescent cells, which increases the stability of active, phosphorylated AKT [43,99].

### Alterations in metabolism

Senescent cells exhibit altered metabolic activity in part due to mitochondrial and lysosomal dysfunction [35,128]. Mitochondria in senescent cells display changes in dynamics and morphology, known as senescence-associated mitochondrial dysfunction (SMAD) [69]. Moreover, due to a reduction in mitophagy function, an accumulation of old and dysfunctional mitochondria can be observed [101]. Mitophagy, the selective autophagy that removes damaged or dysfunctional mitochondria, is essential for maintaining cellular and mitochondrial homeostasis. The inhibition of mitophagy has been linked to cellular senescence and the loss of proliferative capacity in stem cells [17,24,37,69]. The mitochondria are the major site of regulation of reactive oxygen species (ROS) and antioxidant pathways therefore the build-up of damaged mitochondria leads to an increase in ROS production and oxidative stress in senescent cells [122]. Mitochondria dysfunction, in particular of the mitochondrial complex I (NADH: ubiquinone oxidoreductase) in the brain, is associated with cell senescence and ageing [87]. Senescent cells display an upregulation of lysosomal proteins and increased lysosomal content, possibly due to an attempt to balance the build-up of dysfunctional lysosomes [23]. An increase in ROS also targets the lysosomes, inducing more damage and creating a feedback loop deteriorating lysosomal function. The increase in lysosomal mass is linked to the common senescence marker, SA-β-gal activity, while the accumulation of residual lysosomal bodies, named lipofuscin, is also used to identify senescent cells [49,76].

### Changes in proteostasis

The balance between protein synthesis, folding and degradation is vital to protect cells against proteotoxic stress. Cells do so by maintaining the delicate balance between protein production and disposal. Proteostasis is deregulated in senescent cells, promoting endoplasmic reticulum (ER) stress [109]. When cells are stressed, the ER responds by activating the unfolded protein response (UPR). However, evidence suggests that in senescent cells there is deterioration of the UPR, an enlargement of the ER and increased export of misfolded proteins [109]. Moreover, misfolded and aggregated proteins require clearance by autophagy, in senescent cells autophagy is often impaired and induction of autophagy can inactivate senescence [60]. Deregulated proteostasis is also associated to ageing, quantitative analysis of the human adult brain revealed that ageing establishes intrinsic alterations of the proteostasis network which leads to slower protein turnover in the aged adult brain compared to the young adult brain [132]. The maintenance of proteostasis in neurons is especially significant as they are post-mitotic and cannot be readily replaced. A common characteristic of neurodegenerative disease is the presence of proteinaceous inclusions which are composed of misfolded, aggregated and toxic forms of proteins (Yerbury et al., 2016). Therefore, proteostasis is central to healthy ageing and dysregulation of the proteostasis network leads to neurodegenerative disease.

### The SASP

Senescent cells release an elevated level of pro-inflammatory cytokines, chemokines, growth modulators, angiogenic factors and matrix metalloproteinases (MMPs) known as the SASP. In contrast to quiescent cells, in senescent cells the SAGA is accompanied by expression of the SASP [120]. This upregulation of the SASP during senescence is induced by a number of signalling pathways including nuclear factor-kB (NF-κB), p38MAPK, CCAAT/enhancer binding protein-β (C/EBPβ) transcription factors, cyclic GMP-AMP synthase (cGAS), adaptor stimulator of interferon genes (STING) and mTOR signalling [21,56,71,97]. However, activation of NF-κB may also promote survival responses by upregulating anti-apoptotic proteins of the Bcl-2 family [120]. The production and composition of the SASP is highly heterogenous between different senescent cells and varies based on the origin of the stimulus and duration of senescence [55,75]. Among the numerous secreted SASP factors, pro-inflammatory cytokines IL-1a, IL-1β, IL-6 and IL-8 are often released by senescent cells [21,27,56,71,73]. IL-1 and IL-6 mediate senescence in an autocrine manner by reinforcing senescence and pro-inflammation via a cell-autonomous mechanism [2]. Other SASP factors, such as IL-1α, interferon γ (IFNγ), transforming growth factor β (TGF-β) and vascular endothelial growth factor (VEGF) can induce senescence and inflammation in neighbouring cells through a process called paracrine senescence [26]. A small proportion of SASP factors are secreted by exosome-like vesicles, enabling them to exert even more distal spread of senescence [129].

## Senescence and its consequences for the tissue microenvironment

Chronic senescence disrupts tissue homeostasis and becomes maladaptive, particularly in the brain, an organ composed largely of non-proliferative cells that cannot regenerate [45]. In this section we discuss the possible effects of prolonged senescence, focusing on immunosenescence, its link to inflammation and its role in tissue dysfunction and reduced regenerative capacity.

### Immunosenescene and inflammageing

Immunosenescence refers to an age-related decline in innate and adaptive immunity, impairing the clearance of senescent cells and promoting their accumulation. This decline contributes to inflammageing, a chronic inflammatory state linked to tissue degeneration and age-related diseases [80]. Using these mechanisms, senescent cells can propagate senescence to bystander cells, amplify inflammation, and cause systemic dysfunction. For example, senescent cell transplantation in mice induces senescence in surrounding cells, increases inflammation, and causes physical impairments [148]. Conversely, clearing p16-positive senescent cells reduces inflammation and restores immune cell levels to youthful conditions [154].

Additionally, SASP factors can perpetuate their own expression (autocrine signaling) and induce senescence in neighboring cells (paracrine signaling), spreading dysfunction across tissues [98]. Although the immune system initially clears senescent cells, prolonged SASP expression can suppress immune surveillance and promote fibrosis, for example [93]. Thereby, continued SASP exposure creates a positive feedback loop, driving inflammation and further senescence. This process impairs tissue regeneration and contributes to systemic inflammation, particularly in ageing tissues.

In the brain, senescent microglia—the brain’s immune cells—exhibit exaggerated inflammatory responses, releasing SASP mediators such as IL-6, IL-1, TGF-β, and TNF-α. This hyperactivation triggers maladaptive inflammation and recruits peripheral immune cells, compounding chronic inflammation [154]. Neuroinflammation linked to microglial senescence is evident in aged mice and patients with neurodegenerative diseases, which show higher levels of neuroinflammation, microglial over-activation, elevated SASP mediator IL-6, IL-1, TGF-β, TNF-α levels, and an upregulation of the SASP regulator p38MAPK [82].

Interestingly, targeted clearance of senescent cells may alleviate neuroinflammation. For instance, removing p16-positive cells in INK-ATTAC mice reduced microglial activation, normalized cytokine expression, and restored immune cell levels [96]. Similarly, senolytic treatments reversed inflammatory phenotypes in neurodegenerative mouse models, reducing SASP mediators like IL-6 and TNF-α [153].

Senescent cells impair tissue repair and regenerative capacity. In tissues reliant on adult stem cells, such as NSPCs, senescence diminishes regenerative potential and disrupts repair mechanisms [81]. This loss contributes to tissue dysfunction and degenerative diseases [10,96]. Similarly, recent observations demonstrate that senescent NSCs produce their own characteristic SASP (IL-23, Trp53, IL-15, IL-33, and others), which may contribute to disrupting key mechanisms in the preservation of the hippocampal neurogenic niche [47]. This possibility will be discussed in more detail in following sections.

In summary, chronic senescence, driven by immunosenescence, SASP-mediated inflammation and other mechanisms, propagates tissue dysfunction, particularly in the brain. Therefore, targeted strategies to clear senescent cells or mitigate SASP effects hold promise for restoring tissue homeostasis and regenerative capacity in ageing and disease.

### Cellular senescence of NSPCs

The capacity for the adult brain to develop new neurons and glial cells is owed to NSPCs. In the brains of rodents, these adult NSPCs reside in specialised tissue microenvironments termed neurogenic niches, the major ones being the subgranular zone (SGZ) of the dentate gyrus in the hippocampus and the ventricular-subventricular zone (V-SVZ) lining the lateral brain ventricles. While NSPC proliferation is largely restricted to the embryonic stage, within these neurogenic niches, NSPC neurogenesis and gliogenesis can reportedly continue throughout life – a process known as adult hippocampal neurogenesis (AHN) [85], though its existence in humans remains debated [130].

These NSPCs are life-long proliferating cells presumably required to replenish and repair tissues throughout life. However, in their capacity of producing new neurons NSPCs in the adult mammalian brain contribute to cognitive functions and mood regulation. In general, when somatic stem cells undergo senescence, their reduced capacity to proliferate and regenerate contributes greatly to the decline in tissue function. In the brain, this loss of regenerative potential due to a build-up of senescent cells is a major driver of ageing and neurodegenerative diseases [10,45,154]. Although senescence has been observed across various cell types of the brain [25,54,106,119], senescence in NSCPs is less well characterized. Despite limited research, understanding the mechanisms underlying NSPC senescence, may unravel innovative strategies to preserve their proliferative potential and could offer a promising strategy to mitigate the effect of ageing and age-related diseases.

Throughout adulthood, most NSPCs are maintained in quiescence [33]. Unlike in senescent cell cycle arrest, quiescent cells maintain metabolic activity and a high sensitivity to their environment. Diverse physiological stimuli from the adult hippocampal neurogenic niche can promote quiescent NSPCs into undergoing activation, proliferation, migration or fate specification. Quiescence is vital to protect long-lived proliferative cells like adult stem cells from the accumulation of DNA and cellular damage [19]. By lowering molecular, biochemical and metabolic activities, quiescent cells reduce the production of toxic metabolites or DNA replication errors which may contribute to senescence or tumorigenesis [19]. Like senescence, quiescence involves a complex molecular program to supress terminal differentiation, yet in quiescent cells reversibility of the cell cycle arrest is ensured [131]. It is established that active NSPCs undergo replicative senescence due to proliferative exhaustion and an accumulation of cellular damage [94]. However, a hypothesis that is less well investigated is that age-dependent cell-intrinsic alterations take place in a subset of quiescent NSPCs which may switch them from a quiescent to senescent cell fate.

### Transcriptomic/molecular changes

Interestingly, p21, a traditional marker of senescent cells in general, may play a role in modulating the quiescence/activation in NSPCs, as shown by the conditional deletion leading to enhanced activation and expansion of the pool [20]. However, p21 has a dual role in stem cell ageing: it protects adult stem cells from acute genotoxic stress, but it impairs stem cell function and survival in the context of accumulating chronic and persistent damage associated with ageing [63].

Research indicates that significant changes occur in quiescent NSPCs with ageing [7,34,64]. Aged quiescent NSPCs exhibit intrinsic dysfunctions, including impaired proteostasis, reduced autophagy and increased mitochondrial metabolism [58,77,91]. With age, NSPCs showing senescent phenotypes become more prevalent, characterised by an upregulation of key senescence biomarkers such as an upregulation of cell cycle inhibitors p16, p21 and p53, increased activity of SA-β-gal, loss of lamin-B1, DNA damage indicated by γH2AX and the activation of mTOR, DDR and SASP signalling pathways [3,4,62,77,90]. Furthermore, a single cell RNA sequencing comparison between 2 and 4.5 months old quiescent neural stem cells revealed transcriptomic changes indicating early signs of molecular ageing [61]. Changes in processes such as inflammation due to NIK/NF-kappaB signaling, changes in metabolism and proteostasis; cellular stress from loss of DNA recombination, and increased double-strand break repair – have all been associated with senescent cells [61].

Senescent NSPCs can additionally express pro-inflammatory and tissue modulating SASP factors, like IL1α and IL-6, that alter the adult hippocampal neurogenic niche towards pro-inflammation and drive infiltration of systematic innate and adaptive immune cells [39,62]. Notably, a transient surge of circulating IL-6 has been shown to be sufficient for self-renewal and cause short-term proliferation of adult NSPCs in the V-SVZ, ultimately leading to a long-term depletion of the NSPC pool [121]. These findings highlight the dual role of such factors even in regulating NSPC behavior and underscores the need of future studies to investigate the fine-tuning of such pathways.

### Less activation or full proliferative arrest?

Quiescent NSPCs show increased resistance to activation and proliferation and this inactivity may stem from the age-related intrinsic dysfunctions [64]. To this accord, treating aged mice with mTOR inhibitors to boost lysosomal activity can restore the age-driven loss of proteostasis in NSPCs and their proliferative potential [46]. Traditional snapshot-based analysis together with more advanced techniques, like single-cell tracing using intravital imaging in vivo, demonstrate NSPCs shifting to a deeper and long-term quiescence with age [52,61,144]. Moreover, research has identified two distinct NSPC subtypes with varying proliferative potential, morphology, and function [48,83]. As age progresses, NSPCs with a lower proliferative potential become more prevalent, while the number of proliferating NSPCs decreases [83]. This suggests that with age transcriptional and morphological changes occur, favouring a transition of NSPCs to a low proliferative potential [83]. A question that remains to be addressed is if these NSPCs are in a state of deep quiescence or if a subset of them might have turned senescent and are no longer available to replenish the microenvironment with new cells.

### Interplay of NSPCs with their niche

Senescent NSPCs may negatively impact non-senescent cells in their surroundings. Resistance to activation in quiescent NSPCs stems from the age-accelerated inflammatory environment of the niche [64]. Clearance of p16-posititve senescent cells in the SVZ niche of aged mice can reverse neurogenesis decline [90]. Interestingly, the SASP is thought to mediate this paracrine effect, as factors released by senescent neural stem cells inhibit proliferation and promote inflammation in non-senescent counterparts, further impairing brain regeneration [39]. Systematic increase of inflammatory SASP factor CCL11 reduces hippocampal neurogenesis and cognitive functions [134]. Additionally, SASP modulators that activate immunosurveillance may exacerbate neuroinflammation and lead to the decline in neurogenesis [62]. Reportedly, expression of IL-27 by neuroblasts in the dentate gyrus drives NK cell infiltration and activation, which induces inflammation and neurodegeneration [62]. Also, T cells that infiltrate the ageing V-SVZ niche contribute to the resistance of old NSPCs to activation [34]. A recent study further underlines the connection between neuroinflammation and ageing within the DG by demonstrating global transcriptomic changes accompanied by T-cell-mediated inflammatory response and an increase of T cell numbers [146].

Genetic ablation of senescent cells has been shown to improve neurogenesis and hippocampal-dependent cognitive functions, highlighting how an age-related increase of senescent cells can lead to neuroinflammation, reducing NSPC proliferation and hippocampal neurogenesis [1,16,39,90,96,153]. For example, a recent study demonstrated an ageing-dependent accumulation of senescent cells in the hippocampal neurogenic niche, which accompanied the previously described reduction in adult neurogenesis associated with ageing. Interestingly, these senescent cells were in their majority (85 %) neural precursor cells positive for SOX2, while most of the remaining senescent cells were mature astrocytes positive for S100β. Acute pharmacological ablation of these senescent cells promoted neural progenitor activation, neurogenesis and enhanced spatial memory supporting the conclusion that increased cellular senescence is a driver of age-associated cognitive decline [39].The proportion of NSPCs expressing senescent biomarkers varies between studies and is hard to speculate upon due to the differences in experimental conditions. However, in general, senescent cells only make up a small proportion of a tissue [3,4,39,62,90]. Hence, in the young brain, quiescent NSPCs can respond to activation signals to undergo proliferation. However, in the aged brain the NSPC SASP, as well as the accumulation of non-proliferative senescent NSPCs and other senescent cell types, may inhibit the regenerative capacity the adult hippocampal stem cell niche.

## miRNAs as coordinators of NSPC cell fate

miRNAs are a family of small non-coding RNAs that target multiple specific mRNAs and thereby coordinate and regulate a wide array of biological processes [116,118]. Because of their concerted action they are excellent candidates for the coordination of NSPC homeostasis within the adult hippocampal niche [103]. Mechanistically, miRNAs are single-stranded, 19–25 nucleotide long, RNA products that are involved in post-transcriptional regulation and fine tuning of gene expression by downregulation of mRNA targets [12,103]. However, epigenetic regulation of gene expression in NSPCs involves a complex interplay of mechanisms, including DNA methylation, histone modifications, and non-coding RNAs such as miRNAs [103,117,158]. While miRNAs are key epigenetic regulators that influence NSPC proliferation, differentiation and fate determination, they do not act in isolation. Their activity is integrated within broader epigenetic networks, often working in concert with transcription factors and chromatin-modifying enzymes to fine-tune gene expression [117]. There are several intracellular pathways that may mediate NSPC senescence and multiple genes that play roles in this process, as we have discussed in previous sections. This indicates the need of molecular mechanisms that coordinate the orderly progression of senescence. One single miRNA can supress numerous targets involved in multiple signalling pathways, thereby simultaneously regulating distinct cellular processes [123]. In the brain, miRNAs have key roles in neural development, differentiation and maturation. Expectedly, some miRNAs regulate NSPCs quiescence and activation [103,152]. miRNAs may also regulate NSPC senescence and components of the SASP, thereby regulating the inflammatory context that NSPC produce.

### miRNAs in NSPC quiescence and activation

miRNA-9 (miR-9) is a brain-specific miRNA enriched in the neurogenic areas of embryonic and adult brains [31,66,70,74]. Together with nuclear receptor TLX, miR9 forms a regulatory feedback loop which regulates NSPC differentiation and dictates NSPC cell fate [156]. miR-137 is another miRNA associated with NSPC activation and proliferation. miR-137 induces NSPC proliferation in the SGZ by directly targeting the histone methyltransferase and polycomb group protein Ezh2 that regulates cell cycle progression, DNA damage repair and suppression of senescence [86,126]. In addition, miR-132 overexpression in adult mouse NSCs and progenitors in vivo or miR-124 in SVZ NSCs are able to promote neuronal differentiation and maturation [18,102,135]. Moreover, the brain-specific miR-124 can trigger apoptosis-inhibitory pathways by targeting pro-apoptotic BCL-2 proteins [125]. MiR17 ∼ 92 is another cluster highly upregulated in activated neural stem cells, and its conditional deletion in the V-SVZ reduced proliferation [40]. Various other studies have identified a range of miRNAs, effecting the proliferation potential of the NSPC pool, namely let-7b by targeting key factors such as TLX and cyclin D1, miR-25 through its interaction with FOXO3, a downstream factor of the insulin/IGF signalling pathway, and miR-138-5p [14,139,155].

### miRNAs in NSPC senescence

Due to miRNAs’ broad regulatory role in NSPC quiescence and activation, it is possible that aberrant miRNA expression could impact the age-dependent changes that occur to NSPC homeostasis. Recently, reports have suggested that miRNAs also modulate cellular senescence via targeting senescence-associated molecular signalling pathways such as p53-p21, Rb-p16 and those involved in the SASP expression [92]. The miRNAs that are implicated specifically in adult NSPC senescence are yet to be extensively characterized, however profiles of differentially expressed miRNAs in other senescent cell types have been described [123]. For example, miR-34a is upregulated in aged cells and promotes cellular senescence by supressing target SIRT1 [8,89]. The role of miR-34a in NSPC differentiation has been widely studied, yet its part in NSPC senescence has not yet been investigated [6]. There are numerous families of miRNAs that seem to be implicated in the regulatory crossroad between NSPC homeostasis, quiescence and senescence (summarized in Table 1). Further investigation into this subject would be indispensable for depicting the molecular processes that favour the switch of NSPCs to undergo senescence and lead to age-driven brain pathologies. Although NSC senescence was not directly assessed, miR-124 and miR-137 overexpression in the dentate gyrus is associated with downregulation of the proapoptotic Bcl-2 family member BCL2L13, the proapoptotic BAX, and NDUFB6, NDUFB7, two members of the mitochondrial complex I, while proteins with antiapoptotic functions such as Clusterin (CLU) were upregulated. Interestingly, the common miR-124 and miR-137 target BCL2L13 was expressed in NSPCs in the dentate gyrus and controlled caspase 3 expression and activity in NSPCs, strongly indicating that miR-124 and miR-137 cooperativity may impact of NSPC senescence by inhibiting apoptosis [114]. Interestingly, recent work demonstrated that miR-302b can reverse senescence by restoring the proliferative capacity of senescent cells. When delivered in vivo to ageing mice using human embryonic stem cell-derived exosomes, miR-302b promoted extended lifespan, improved physical performance and reduced ageing markers, demonstrated the capacity of miRNAs to mitigate senescence-related pathologies and ageing [13].

**Table 1:** MiRNAs at the crossroad of neural stem/progenitor cell (NSPC) homeostasis and senescence. This table summarizes key miRNAs involved in the regulation of NSPC homeostasis, with a focus on their roles in NSPC proliferation, differentiation, and which also have been implicated in regulating aspects of cellular senescence.

miRNAs	Identified targets	Role in NSPC homeostasis	Role in senescence	References
miR-34a	SIRT1	NSPC differentiation	Activation of p53/p21 and SASP pathway	[6], [8], [89]
miR-17–92 cluster (miR-17, 19b, 20a, 106a)	p21, ADCY5, IRS1, Bim, HIF-1a	NSPC activation/ proliferation, and neural differentiation	Represses PRAS40 in mTOR pathway	[163], [164], [165], [166]
miR-124	BCL2L13, SOX9, RyR3, PTPN1	NSPC differentiation	Target pro-apoptotic BCL-2 proteins	[18], [114], [125]
miR-132	PTEN, FOXO3, Nurr1, SIRT1	NSPC neurogenesis	Supresses AKT signalling	[103], [135]
let-7	TLX, HMGA2, p53 signalling components (p66Shc)	NSPC renewal	Targets downstream p53 signalling, overexpression results in premature senescence	[139], [155]
miR-146a/b	IRAK, TRAF6, TLR2, CRCX4	NSPC differentiation	SASP signalling	[35], [103], [127]

### miRNAs in SASP regulation

Another way in which miRNA can influence inflammation and senescence in the brain is via the regulation of SASP signalling pathways and specific SASP factors. For instance, miR-146a is a known inhibitor of the NF-κB pathway, by supressing the upstream activators tumor necrosis factor receptor-associated factor 6 (TRAF6) and interleukin-1 receptor-associated kinase-1 (IRAK1), mitigating SASP-associated inflammation [36,127]. In addition, miR-9 has also been shown to downregulate NF-κB signaling, reducing SASP-related inflammatory cytokines in microglia [149]. Various other miRNAs (e.g. miR-143, miR-187, miR-222, and miR-125b) modulate the secretion of cytokines, growth factors and proteases [92]. However, a direct assessment of miRNA-mediated SASP regulation in brain tissue, especially in the adult neurogenic niches, is still lacking.

## miRNAs in neurodegenerative diseases and neuroinflammation

Various studies have indicated that miRNAs play a pivotal role in the regulation of genes and molecular pathways associated with neurodegenerative diseases like AD [18], [36], [68], [103], [114], [125], [127], [136], [152]. MiRNA-based therapeutics offer a unique and powerful avenue for gene network-based approaches because of the inherent ability of miRNAs to simultaneously regulate multiple target genes within interconnected signaling pathways. Unlike traditional therapies that may typically target single genes or proteins, miRNAs can fine-tune entire gene expression programs by binding to common complementary sequences in the 3′ untranslated regions (3′ UTRs) of multiple target mRNAs. This allows them to modulate complex cellular processes such as proliferation, and inflammation [137]. For example, miR-132 is one of the most abundant miRNAs in the brain and is implicated in various neurophysiological and pathophysiological processes [68,152]. In AD brains, miR-132 is the most downregulated miRNA and its downregulation is thought to be involved in the progression of Aβ and tauopathy [152]. Emerging evidence suggests that miR-132 also has anti-inflammatory roles in vitro and in vivo [136]. Other miRNAs that are involved in senescence, such as miR-124 and miR-146 have also been implicated in neurodegeneration through their regulation at the interface of neuronal aspects and the immune system (Table 1). This supports that research into miRNAs may be key to uncovering the complex regulatory mechanisms that underlie AD and other neurodegenerative diseases.

### Could miRNAs be used as senotherapeutics for neurodegenerative diseases?

Ageing is the strongest known risk for the development of neurodegenerative diseases [59]. As introduced in previous sections, senescent cells accumulate in tissues throughout healthy ageing and also contribute to a plethora of age-related pathologies [9,11,16,53,96,148]. The brain, and the hippocampus in particular, may be especially susceptible to this build-up of senescent cells, due to its sensitivity to the pro-inflammatory environment that senescent cells create [15,38,42]. This link between cellular senescence in the initiation and propagation of age-related neurodegeneration had sparked interest in senotherapeutics − strategies to interfere with the detrimental effects of senescent cells [78,151]. Up to know, senotherapeutics can be classified into two generations, which include senolytics (agents that eliminate senescent cells) and senomorphics (agents that modify the senescent secretome).

Given the detrimental effects associated with accumulation of senescent cells with aging and its possible association with neuroinflammations, several therapeutics strategies have been experimentally developed, and their potential applications are currently being evaluated. 1) Delivery of suicide genes for gene therapy: for example, clearance of senescent astrocytes and microglia expressing the cell cycle inhibitory protein p16^INK4A^ in INK-ATTAC transgenic mice [11] prevents gliosis, hyperphosphorylation of both soluble and insoluble tau, neurofibrillary tangle deposition, degeneration of cortical and hippocampal neurons, and preserved cognitive function [16]. Similarly, CRISPR/Cas9-mediated gene correction approaches have been applied to rescue stem cells from premature aging [140]; 2) Pharmaceutics approaches: senolytic drugs such as Dasatinib, Quercetin, Navitoclax (ABT-263), Fisetin, FOXO4-DRI Peptides and HSP90 Inhibitors have been developed to target senescent cells [78]. Senolytics target the anti-apoptotic pathways of senescent cells to induce cell death [67,78]. On the other hand senomorphics supress the signalling pathways that contribute to the SASP by targeting mTOR, NF-κB, IL-1α and p38MAPK signalling pathways [5,88]. For example, rapamycin is a well-established, FDA-approved senomorphic. Rapamycin is an inhibitor of mTOR, leading to the suppression of IL-1α production and the transcription of inflammatory genes by NF-κB [143]. It has been demonstrated that rapamycin can increase lifespan of mice, as well as alleviate age-related dysfunctions [79]. Besides rapamycin, other senolytics and senomorphics are currently under investigation in preclinical but also in clinical settings, yielding promising therapeutic effects for alleviating neurodegenerative diseases [141]. Moreover, next-generation senolytic therapeutics with desired disease and tissue specificity, such as immunological approaches (CAR-T cells, Antibody-Drug Conjugated or vaccines), are being developed [78]. However, there are significant challenges associated with the clinical implementation of senescent cell targeting. Senescence markers may not be exclusively expressed in senescent cells and targeting them could resulting in the unwanted depletion of beneficial cell populations [60]. In addition, some of the first generation senolytics such as Navitoclax can cause alterations in white blood cell formula by promoting apoptosis in non-senescent cells [30]. Finally, future specific strategies such as gene therapy or immunological approaches face well-known issues regarding off-targeting and delivery [78], specifically to the brain.

Given the complexity of neurodegenerative diseases, a single-target approach might be ineffective, and the focus should shift to “multiple drugs – multiple targets” approaches. In this regard miRNA-based therapeutics might offer a unique avenue for a more gene network-based approaches, as they regulate common sensitive hubs within the central nervous system and are deregulated with age and in age-related disorders, such as AD [108,135]. Indeed, although its effects on the brain were not reported, miR-302b delivery in vivo to ageing mice reverted cellular senescence, restored proliferation and reduced inflammation hallmarks in the liver, kidney, lung and skin, highlighting the potential of miRNAs as future senotherapeutics [13]. Evidence suggests that miRNAs are master regulators of the balance between NSPC quiescence, neurogenesis, and other fates. As discussed earlier, miRNAs have also been implicated in the signalling pathways relating to senescence [103,123,152]. miRNA-based therapeutics mainly consist of synthetic miRNAs which increase miRNA levels back to normal, or antisense oligonucleotides which inhibit the endogenous miRNAs [135]. For example, elevating miR-132 levels, which is downregulated in AD, aggravates AD pathology, aspects of inflammation, and restores hippocampal neurogenesis is the adult AD mouse brain [111,112,136]. Alternatively, inhibition of a senescence-promoting miRNA, such as miR-34c may be sufficient to reduce neurodegeneration [8,89]. Therefore, it may be possible to reduce the negative consequences of senescent cell build-up on the brain by inhibition of miRNA activated senescence signalling pathways in ageing brain cells. Evidence demonstrates that miR-34c levels are elevated in the hippocampus of AD patients and mouse models and targeting miR-34c can rescue learning ability in mouse models [159]. Although the group did not investigate whether targeting miR-34 reduced cellular senescence in the mouse brains, one could hypothesise that SIRT1, a target of miR-34, would be more abundant to inhibit senescent pathways [142]. This idea warrants more research into the miRNAs specifically responsible for senescence in the brain and particularly in the hippocampus but could serve as a foundation to explore miRNAs as senotherapeutics, combinatorial with their effect to combat neurodegenerative diseases. However, this approach will face its own challenges, as delivering therapeutics to the brain requires overcoming the blood–brain barrier (BBB) that restricts the passage of most molecules from the bloodstream into the brain tissue. While essential for maintaining neural homeostasis and protecting against toxins and pathogens, the BBB also limits the entry of many potentially beneficial drugs, especially large or hydrophilic molecules such as miRNAs. Some promising strategies have been designed to overcome the BBB such as nanoparticle carriers, viral vectors, receptor-mediated transport systems, or direct delivery methods like intracerebroventricular injection [145]. However, these approaches should balance efficacy, safety, specificity and the ability to avoid immune activation or other off-target effects [57].

## Future directions

The future development and clinical application of senotherapeutic drugs faces several challenges that must be addressed before their therapeutic potential can be fully developed. Many current senotherapeutic drugs target only a single pathway involved in cellular senescence, limiting their efficacy across diverse cell types and tissues. Moreover, some compounds may affect healthy, non-senescent cells, raising concerns about potential off-target effects. In this context, long-term administration could lead to unintended side effects or toxicity. A major hurdle remains the heterogeneity of senescent cells, which vary widely in their molecular profiles depending on tissue type, ageing stage, and underlying pathology. Addressing these challenges may requires multi-targeted therapy approaches. Therefore, cell-type specific delivery of senotherapeutic miRNAs using viral vectors and specific promoters may provide a suitable and precision medicine strategies to create safer, more effective, and broadly applicable senotherapeutics.

## Credit authorship contribution statement

**O. Polzer:** Writing – review & editing, Writing – original draft, Visualization, Conceptualization. **E. Kinloch:** Writing – review & editing, Writing – original draft, Visualization. **C.P. Fitzsimons:** Writing – review & editing, Writing – original draft, Visualization, Validation, Supervision, Project administration, Funding acquisition, Conceptualization.

## Declaration of Generative AI and AI-assisted technologies in the writing process

During the preparation of this work the author(s) have occasionally used ChatGPT only to improve language and readability. After using this tool/service, the author(s) reviewed and edited the content as needed and take(s) full responsibility for the content of the publication.

## Declaration of competing interest

The authors declare that they have no known competing financial interests or personal relationships that could have appeared to influence the work reported in this paper.
